# Activity of Binary Combinations of Natural Phenolics and Synthetic Food Preservatives against Food Spoilage Yeasts

**DOI:** 10.3390/foods12061338

**Published:** 2023-03-22

**Authors:** Bernard Gitura Kimani, Miklós Takó, Csilla Veres, Judit Krisch, Tamás Papp, Erika Beáta Kerekes, Csaba Vágvölgyi

**Affiliations:** 1 Department of Microbiology, Faculty of Science and Informatics, University of Szeged, Közép fasor 52, H-6726 Szeged, Hungary; 2 Department of Food Engineering, Faculty of Engineering, University of Szeged, Mars tér 7, H-6724 Szeged, Hungary; 3 ELKH-SZTE Fungal Pathogenicity Mechanisms Research Group, University of Szeged, Közép fasor 52, H-6726 Szeged, Hungary

**Keywords:** checkerboard method, combined effect, growth inhibition, phenolic compounds, spoilage yeasts, synthetic preservatives

## Abstract

Natural compounds are a suitable alternative to synthetic food preservatives due to their natural origin and health-promoting properties. In the current study, phenolic–phenolic and phenolic–synthetic combinations were tested for their antibiofilm formation, anti-planktonic growth, and anti-adhesion properties against *Debaryomyces hansenii*, *Wickerhamomyces anomalus* (formerly *Pichia anomala*), *Schizosaccharomyces pombe*, and *Saccharomyces cerevisiae*. The phenolics were vanillin and cinnamic acid, while the synthetic preservatives were sodium benzoate, potassium sorbate, and sodium diacetate. The vanillin–cinnamic acid combination had synergistic effect in all the tested yeasts for the biofilm inhibition with a fractional inhibitory concentration index (FICI) of ≤0.19 for *W. anomalus*, 0.25 for *S. pombe*, 0.31 for *S*. *cerevisiae*, and 0.5 for *D. hansenii*. Most of the phenolic–synthetic combinations had indifferent interaction regarding biofilm formation. The vanillin–cinnamic acid combination also had higher activity against spoilage yeasts adhesion on the abiotic surface and planktonic growth compared to the phenolic–synthetic combinations. For the phenolic–synthetic anti-planktonic activity, synergistic interaction was present in all the vanillin–synthetic combinations in *S. pombe*, vanillin–sodium benzoate and vanillin–potassium sorbate in *S. cerevisiae*, vanillin–sodium benzoate in *W. anomalus*, and cinnamic acid–sodium diacetate in *S. pombe*. These results suggest a novel antimicrobial strategy that may broaden the antimicrobial spectrum and reduce compound toxicity against food spoilage yeasts.

## 1. Introduction

Yeasts are an integral part of the Earth’s ecosystem, known and used since antiquity in the production and development of valuable products such as ethanol, probiotics, pigments, and pharmaceutical products [1]. While accepted as an illustrious microbe that has positively impacted food and beverage production since the dawn of humanity, some yeasts also play a role in food spoilage, causing massive economic losses due to their high resilience in adverse conditions [2]. Yeast spoilage of foods may impair food quality, make nutrients unavailable, and affect food safety [3,4]. Some *Zygosaccharomyces* and *Debaryomyces* (e.g., *Debaryomyces hansenii*) species are osmotolerant and halotolerant and may spoil honey and dried fruits [5]. *Wickerhamomyces anomalus* (formerly known as *Pichia anomala*) is an ascomycetous heterothallic yeast that can grow at a broad pH range, high osmotic pressure, and low water activity, and is hence capable of spoiling high-sugar content foods [6]. Some yeast species contaminate fruit salads and juices, leading to spoilage through discoloration and production of off-flavors [7]. In fact, continuous attention should be paid to preventive strategies that can reduce the activity of spoilage yeasts in foods and are accepted as an ecofriendly approach by the consumers.

The use of chemical-based food preservatives in food production processes remains the main strategy to control microbial food spoilage due to their low cost and strong antimicrobial activity [8]. Though effective at inhibiting microbial proliferation in fresh and stored foods, consumers’ demand for high-quality foods with little or no chemical residue has led to a search for a safe and environmentally friendly alternative [9]. Chemical food preservatives become toxic when ingested above the acceptable daily intake [10]. The addition of potassium sorbate, sodium acetate, sodium benzoate, and other chemical additives during food processing is crucial for flavor and texture enhancement in addition to their antimicrobial effects [11]. However, high consumption of salts may lead to hypertension and cardiovascular diseases [10,12]. It is believed that sodium benzoate can be decarboxylated in the presence of vitamin C into benzene, a compound of high toxicity and teratogenicity [13]. Sodium benzoate is also implicated in hormonal disruption, generation of oxidative stress, and reduced fertility, and it may influence tryptophan metabolism [10,13]. A recent study reported distinct signatures of gut microbiota on mice consuming potassium sorbate [14].

The use of natural compounds as food preservatives is gaining traction lately due to the increasing interest in minimally processed foods and the desire to decrease chemical food preservatives [15]. Natural food preservatives are available from several sources, including plants, animals, and microorganisms [16]. Plant-derived preservatives include essential oils and phenolic compounds, animal-derived preservatives include chitosan from animal shells, and microbial preservatives include bacteriocins [16]. These natural products have demonstrated great antimicrobial potential against food spoilage [17]. Natural phenolics are ubiquitous compounds that form the major components of plants’ defense systems. Phenolics’ appealing properties, such as the antimicrobial and antioxidant activities, including their nutritive and pharmacological benefits, are well established and are of great scientific interest [18]. These bioactive compounds have been applied in innovative ways in diverse fields, such as in aquaculture [18], as biostimulants and bioprotectants [19], in the cosmetic industry [20], in the improvement of sport performances [18], and in food preservation [21]. The antimicrobial properties of natural phenolics have received widespread attention due to the potential negative health impact of the chemical antimicrobial agents [22]. However, the natural phenolic additives may result in sensorial attributes in certain foods unacceptable to consumers [23]. The presence of synthetic additives in combination with natural phenolics could help mitigate such unpalatable properties, thus preventing a complete loss of the native food taste [8,24].

The application of antimicrobials in combination for food preservation may be more advantageous than singular antimicrobials because the mixture could have synergistic or additive effects [24]. An efficient antimicrobial combination selection is a non-trivial task due to the multiple combinations possible [25]. A good in vitro estimation of the concerted effects of the combined compounds can be determined using the checkerboard method [26]. From the optical density results obtained, the percentage growth per well can be calculated, and the minimum inhibitory concentration (MIC) of the combinations determined. Such combinations may lead to different phenotypic effects, such as synergistic, additive, indifferent, or antagonistic interactions, regarding the growth inhibition [25]. The combination results can be expressed as a fractional inhibitory concentration index (FICI) [26]. There are few data on the effects of binary combinations of antimicrobials against food spoilage yeasts. The present study aimed to elucidate the effect of phenolic–phenolic and phenolic–synthetic preservative combinations against the planktonic and biofilm growth and the adhesion capacity of *D. hansenii*, *W. anomalus*, *Schizosaccharomyces pombe*, and *Saccharomyces cerevisiae* spoilage yeasts using the checkerboard method. Two phenolic compounds, i.e., vanillin and cinnamic acid, were selected for the study because they demonstrated high inhibitory properties against planktonic and biofilm growth of spoilage yeast in a previous investigation [27]. These bioactive compounds belong to different classes of natural phenolics, vanillin being a phenolic aldehyde, while cinnamic acid is a hydroxycinnamate. The synthetic preservatives used in the assay were sodium benzoate, potassium sorbate, and sodium diacetate.

## 2. Materials and Methods

### 2.1. Yeast Strains and Growth Conditions

Four food spoilage yeasts, namely, *D. hansenii* SZMC 8045Mo, *W. anomalus* SZMC 8061Mo, *S. cerevisiae* SZMC 1279, and *S. pombe* SZMC 1280 were used in the assays. All the yeast strains were obtained from the Szeged Microbiological Collection (SZMC) maintained by the Department of Microbiology, University of Szeged, Hungary (http://szmc.hu/, accessed on 3 February 2023). *W. anomalus*, *S. cerevisiae*, and *S. pombe* were grown on a malt extract (ME) medium containing 5 g/L yeast extract (Biolab, Budapest, Hungary), 5 g/L glucose (Biolab, Budapest, Hungary), and 50 mL/L 20% (*v/v*) malt extract (Merck, Budapest, Hungary). *D. hansenii* was cultivated on yeast extract peptone dextrose (YPD) medium containing 20 g/L glucose (Biolab, Budapest, Hungary), 20 g/L peptone (Sigma-Aldrich, Munich, Germany), and 10 g/L yeast extract (Biolab, Budapest, Hungary). Before each assay, fresh yeast cultures were prepared in 20 mL of the corresponding growth medium and incubated for 24 h at 30 °C. At the end of the incubation period, the growth of the yeast was in the stationary phase. After preparing a 10-fold serial dilution with the corresponding growth medium, the cell number was set to 10^6^ CFU/mL by counting in a Bürker chamber under a light microscope.

### 2.2. Phenolic Compounds and Synthetic Preservatives

The two phenolic compounds used in the study, i.e., vanillin and cinnamic acid, and the synthetic compounds, i.e., sodium benzoate, potassium sorbate, and sodium diacetate, were purchased from Sigma-Aldrich (Munich, Germany). The phenolic compounds were selected based on a previous investigation that indicated very high antimicrobial activity of the two natural compounds against food spoilage yeasts [27]. The stock solutions of the phenolics and synthetic compounds were prepared in 10% (*v/v*) ethanol.

### 2.3. Determination of Minimum Inhibitory Concentration (MIC)

The MICs of vanillin and cinnamic acid against planktonic growth of the tested yeasts were reconfirmed using the microdilution technique described previously [27]. The MIC values of the synthetic preservatives on planktonic yeasts were assayed through a microplate method previously described by Zambrano et al. [28]. Briefly, stock solutions of synthetic preservatives were serially diluted with 10% (*v/v*) ethanol from 100 mg/mL to 0.78125 mg/mL concentration. A volume of 100 µL from the diluted samples and the corresponding stock solution were transferred to the wells of a 96-well polystyrene microtiter plate (Sarstedt, Nümbrecht, Germany). A volume of 100 µL of cell suspension (10^5^ CFU/mL) prepared in a double concentration medium was then added to each well, giving a final concentration of 50 mg/mL to 0.390625 mg/mL for the compounds under investigation. Positive controls contained the inoculated growth medium without the synthetic preservative, while the negative controls had the synthetic preservative in sterile medium. After 24 h incubation at 30 °C, absorbance was measured at 600 nm using a SPECTROstar Nano (BMG Labtech, Offenburg, Germany) microplate reader. The concentration of the synthetic preservative that caused 90% or higher growth inhibition compared to the positive control was considered as the MIC.

The MIC of the synthetic and phenolic compounds on yeasts in a biofilm form was determined as follows: 200 µL of 24 h old yeast culture with approximately 10^8^ CFU was pipetted in the wells of a 96-well microtiter plate and incubated for 4 h for cell adhesion at 30 °C. After the incubation, the non-adhered planktonic cells were removed from each well, and the plates were rinsed with physiological saline and left to dry for 10 min. After drying, 100 µL of the compounds were dispensed in the wells followed by the addition of another 100 µL of two-fold concentrated sterile medium to have a final concentration of 64 to 1 mg/mL for the synthetic and 32 to 1 mg/mL for the phenolic compounds. The positive control had the adhered cells and the sterile medium, while the negative control had the compounds and the sterile medium without the adhered cells. The plates were incubated for 24 h at 30 °C, after which biofilms were detected by crystal violet staining assay, as described by Kerekes et al. [29]. The concentration that caused 90% or higher biofilm inhibition compared to the positive control was considered as the MIC.

In adhesion inhibition tests, the MIC for the antimicrobials was determined as follows: the synthetic compounds were pipetted into microtiter plates to achieve a final concentration of 64 to 1 mg/mL, while the phenolics were dispensed in the wells to achieve a final concentration of 32 to 1 mg/mL after the inoculum addition. The positive control had the sterile medium and the inoculum, while the negative control had the sterile medium and the compounds only. The plates were incubated for 4 h at 30 °C, after which the adhered cells were detected by crystal violet staining, as described by Kerekes et al. [29]. The concentration of the synthetic preservative that caused 90% or higher adhesion inhibition compared to the positive control was considered as the MIC.

### 2.4. Checkerboard Assay for Planktonic Growth Inhibition

To evaluate the antimicrobial effect of the phenolic–phenolic and the phenolic–synthetic combinations against the planktonic growth of food spoilage yeasts, the checkerboard method was used, as described by Motyl et al. [30], with minor modifications. The assay was performed using a 96-well polystyrene microtiter plate. Briefly, seven serial two-fold dilutions of the synthetic compounds and six serial two-fold dilutions of the phenolics were prepared. In a 96-well plate, 50 μL of each dilution of the synthetic compounds was dispensed in each vertical row, and 50 μL of the phenolic compound dilution was dispensed in each horizontal row. The selection of the range of concentrations was based on the MICs obtained for the tested compounds against the spoilage yeasts. The final concentration of the synthetic preservatives after microdilution ranged from 64 to 1 mg/mL, while that of the phenolic compounds ranged from 4 to 0.125 mg/mL. The final concentration of the inoculum in each well was 10^5^ CFU/mL. After 24 h incubation at 30 °C under static conditions, growth in each well was quantified spectrophotometrically at 600 nm using a SPECTROstar Nano (BMG Labtech, Offenburg, Germany) microplate reader. Wells that contained only the growth medium and the inoculum without the antimicrobial agents formed the positive control, while the negative control had the sterile medium with the antimicrobials without the inoculum. The percentage of growth in each well was calculated as previously reported [31]. The MIC for each combination was defined as the concentration of compounds that reduced growth by 90% and above compared to that of the yeasts grown in the absence of the antimicrobials. The fractional inhibitory concentration index was computed using the following equation:

FICI = FIC-A + FIC-B = (MIC-AB/MIC-A) + (MIC-BA/MIC-B)

where A and B are the antimicrobial compounds under combination. The FICI is the fractional inhibitory concentration index, FIC-A is the fractional inhibitory concentration of compound A, FIC-B is the fractional inhibitory concentration of compound B, and MIC-AB is the MIC of compound A in the presence of compound B. MIC-BA is the MIC of compound B in the presence of compound A. The interaction was interpreted as synergistic if the FICI ≤ 0.5, additive when 0.5 < FICI ≤ 1, indifferent when 1 < FICI ≤ 4, and antagonistic when FICI > 4.0 [24,32].

### 2.5. Checkerboard Assay for Biofilm Formation Inhibition

To evaluate the antibiofilm effects of the phenolic–phenolic and phenolic–synthetic combinations, the compounds that had FICI < 1 in the planktonic growth inhibition were sampled for the antibiofilm assay. The wells of a 96-well polystyrene microtiter plate were filled with 200 µL of 24 h yeast culture with approximately 10^8^ CFU, except for those wells that formed the negative control. After 4 h of cell adhesion at 30 °C, the planktonic cells were removed from each well, and the plates were rinsed with physiological saline and left to dry in a laminar flow for 10 min. After drying, the synthetic compounds were dispensed in the wells as described in Section 2.4, while the phenolics had a final concentration range of 32 to 1 mg/mL. The positive and the negative controls were as described in Section 2.4. The prepared plates were incubated for 24 h at 30 °C, after which biofilm formation was detected by crystal violet staining as described by Kerekes et al. [29]. The FIC index calculation and the definition of the interactions were as described in Section 2.4.

### 2.6. Checkerboard Assay for Adhesion Inhibition

The combinations used in the antibiofilm assay were also evaluated for their ability to inhibit spoilage yeasts adhesion on a polystyrene surface. The compounds were dispensed as described in Section 2.5, followed by the addition of the inoculum to achieve a final cell count of 10^8^ CFU. The positive and the negative controls were as described in Section 2.4. Plates were incubated for 4 h at 30 °C, after which the adhered cells were detected by crystal violet staining [29]. The FIC index calculation and the definition of the interactions were as described in Section 2.4.

### 2.7. Statistical Analysis

Assays were performed in at least three independent experiments, and the data obtained were expressed as means ± standard deviation. Means and standard deviations were calculated using Microsoft Office Excel 2016 function. Significance was calculated by one-way analysis of variance (ANOVA) followed by Tukey’s multiple comparison test in the GraphPad Prism 6.00 software (GraphPad Software Inc., San Diego, CA, USA). A *p*-value of <0.05 was considered as statistically significant.

## 3. Results

### 3.1. Effects of Combination of Antimicrobial Agents on Planktonic Growth of Yeasts

Vanillin and cinnamic acid were separately combined with sodium benzoate, potassium sorbate, and sodium diacetate synthetic food preservatives to create bipartite solutions that were screened for their anti-yeast activity. The vanillin–cinnamic acid combination was also screened for its efficacy in inhibiting planktonic growth of the food spoilage yeasts. Results for the MIC reduction fold and FICI for the vanillin–cinnamic, vanillin–synthetics, and cinnamic acid–synthetics combinations, are summarized in Table 1, Table 2, and Table 3, respectively. In the vanillin–cinnamic acid combination assays, a synergistic interaction, together with considerable growth inhibition, was identified in all the combinations (Figure S1). For *S. pombe* and *S. cerevisiae*, the FICI was ≤0.28 (*p* < 0.05), while for *D. hansenii* and *W. anomalus*, it was ≤0.31 (*p* < 0.05) (Table 1). The MIC of vanillin was reduced by 32-fold in *S. pombe* and *S. cerevisiae* and by 16-fold in *D. hansenii* and *W. anomalus* when the two phenolics were applied in combination. In this context, a reduction in the MIC of cinnamic acid was also detected (Table 1).

For the phenolic–synthetic combinations, synergistic interaction was present in all the vanillin–synthetic combinations in *S. pombe* (Table 2). A synergism effect was also present between vanillin–sodium benzoate and vanillin–potassium sorbate in *S. cerevisiae* and vanillin–sodium benzoate in *W. anomalus* (Table 2). Checkerboard layouts depicting the activity of some effective phenolic–synthetic combinations against the planktonic growth of the spoilage yeasts tested are shown in Figure S2.

For the cinnamic acid–synthetic combinations, synergism effect was only present between cinnamic acid–sodium diacetate in *S. pombe* (Table 3). There was less antimicrobial activity against *D. hansenii* in all the phenolic–synthetic combinations compared to the other three spoilage yeasts (Table 2 and Table 3). Cinnamic acid–sodium benzoate and cinnamic acid–sodium diacetate were antagonistic in interaction against *D. hansenii* growth (Table 3).

**Figure 1:**
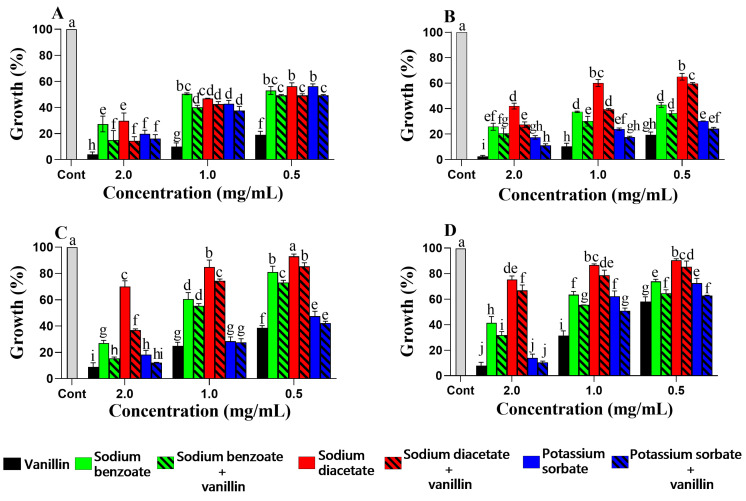
Effect of the antimicrobial agents on planktonic growth of *D. hansenii* SZMC 8045Mo (**A**), *W. anomalus* SZMC 8061Mo (**B**), *S. pombe* SZMC 1280 (**C**), and *S. cerevisiae* SZMC 1279 (**D**) when the vanillin and synthetic agents were used alone or in combination in equal proportions. The control (Cont.) represents growth in the absence of the compounds. The results are the mean percent growth relative to the control; error bars represent standard deviation. The different letters above the columns indicate statistically significant differences (*p* < 0.05).

**Figure 2:**
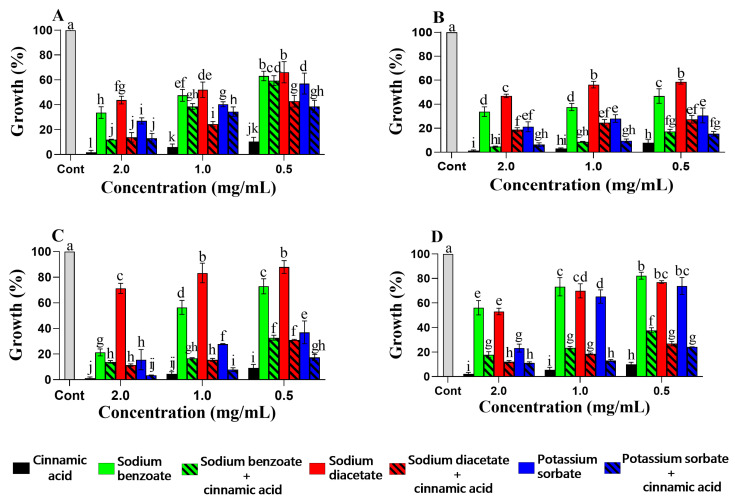
Effect of the antimicrobial agents on the planktonic growth of *D. hansenii* SZMC 8045Mo (**A**), *W. anomalus* SZMC 8061Mo (**B**), *S. pombe* SZMC 1280 (**C**), and *S. cerevisiae* SZMC 1279 (**D**) when the cinnamic acid and synthetic agents were used alone or in combination in equal proportions. The control (Cont.) represents growth in the absence of the compounds. The results are the mean percent growth relative to the control; error bars represent standard deviation. The different letters above the columns indicate statistically significant differences (*p* < 0.05).

Taken together, when equal concentrations of vanillin/cinnamic acid and synthetic preservatives were considered (see Figure 1 and Figure 2), it can be observed that the phenolic–synthetic combinations had generally higher planktonic growth inhibition than the synthetic compounds alone, indicating that the addition of the phenolic compound even at low concentrations had a positive effect on the antimicrobial properties of the mixture. In most phenolic–synthetic combinations, the phenolics had a reducing effect on the MIC of the synthetic additives (Table 2 and Table 3). In *D. hansenii*, for instance, there was a 50-fold reduction in the MIC of sodium benzoate in the presence of vanillin (Table 2). In *S. cerevisiae*, potassium sorbate and sodium benzoate had a 10.7-fold reduction in their MIC when combined with vanillin (Table 2). Combinations of vanillin–sodium benzoate and cinnamic acid–sodium benzoate in *W. anomalus* and cinnamic acid–sodium diacetate in *S. pombe* resulted in a 12.5-fold reduction in the MIC of the corresponding synthetic compound (Table 2 and Table 3). It is worth mentioning that the MIC values of vanillin and cinnamic acid increased in some combination tests compared to those obtained in their single application (Table 2 and Table 3).

### 3.2. Effects of Combination of Antimicrobial Agents on Biofilm Formation

The phenolic–synthetic and phenolic–phenolic combinations were also tested against the biofilm formation of spoilage yeasts. For the phenolic–synthetic combinations, only the combinations with less than 1 FIC index for the planktonic growth were subjected to the biofilm inhibitory assay. As shown in the checkerboard layouts, both the phenolic–synthetic preservative (Figure S3) and the vanillin–cinnamic acid (Figure S4) combination assay resulted in effective combinations against each yeast biofilm tested. Based on the FIC index results, there was an indifferent compound interaction outcome for most phenolic–synthetic combinations in anti-biofilm tests (Table 4). However, the MIC values of many synthetic preservatives were considerably reduced in the presence of phenolics. In *W. anomalus*, for instance, sodium benzoate and potassium sorbate had a 16-fold MIC reduction in the presence of vanillin (Table 4). In the case of *S. pombe*, sodium benzoate had a 64-fold MIC reduction with cinnamic acid and a 32-fold reduction when combined with vanillin (Table 4). A 32-fold MIC reduction was measured for both sodium benzoate and potassium sorbate for *S. cerevisiae* in the presence of vanillin (Table 4).

The phenolic–phenolic combination was synergistic in the case of all yeasts tested with an FIC index of ≤0.5 (*p* < 0.05) (Table 5). The two phenolics also influenced each other’s MIC with a reduction fold of ≥4 (Table 5).

### 3.3. Effects of Combination of Antimicrobial Agents on Adhesion Capacity

The antimicrobial combinations used in the biofilm assay were also evaluated for their ability to inhibit the adhesion of the tested yeasts on a polystyrene surface. Synergistic interaction was observed between vanillin and potassium sorbate in the case of *W. anomalus* and *S. pombe*, with FIC index values of 0.25 and 0.38, respectively (*p* < 0.05) (Table 6). In *S. pombe*, most phenolic–synthetic combinations were additive with the FIC index ranging from 0.53–0.75 (*p* < 0.05) while for *S. cerevisiae*, all the phenolic–synthetic combinations were indifferent in interaction, with the FIC index ranging from 1.5 to 2.5 (*p* < 0.05) (Table 6). In *W. anomalus*, except for the vanillin–potassium sorbate combination, the rest were indifferent in interaction for adhesion inhibition (Table 6). The presence of phenolics had a reducing effect on the MIC value of the synthetic preservatives used in most strains. In *W. anomalus*, for instance, sodium benzoate and potassium sorbate had a 32-fold and 8-fold reduction in MIC in the presence of vanillin (Table 6). In *S. pombe*, sodium diacetate had a 32-fold reduction in the MIC when vanillin was added to the growth medium, while sodium benzoate had a 16-fold MIC reduction with cinnamic acid. In *S. cerevisiae*, potassium sorbate had a 16-fold MIC reduction when combined with vanillin. The overall reduction in the MIC value of synthetic preservatives in the presence of phenolics was between 1 and 32-fold (*p* < 0.05) (Table 6).

The vanillin–cinnamic acid combination was synergistic in interaction in all the yeasts tested, except in *D. hansenii,* where an additivity effect was observed (Table 7). The combination of these phenolic compounds also allowed the use of reduced concentrations, showing a 2- to 16-fold MIC reduction (*p* < 0.05).

## 4. Discussion

The investigation of the interaction between antimicrobial agents can provide information for developing new anti-yeast strategies for enhancing food safety. In fact, several antimicrobial compounds may become more efficient inhibitors in combined application than when used as single agents [33]. The combination may broaden the spectrum of activity of individual compounds, reduce their toxicity, lower their effective dosages, and reduce the chances of antimicrobial resistance [32,33,34].

In this study, vanillin–cinnamic acid and vanillin/cinnamic acid–sodium benzoate/potassium sorbate/sodium diacetate synthetic preservative combinations were tested for their anti-planktonic growth, antibiofilm, and antiadhesion properties against the food spoilage yeasts *D. hansenii*, *W. anomalus*, *S. pombe*, and *S. cerevisiae*, using a checkerboard approach. In the vanillin–cinnamic acid combination, the FIC indices for the planktonic growth inhibition were ≤0.31 (*p* < 0.05), indicating that the combination was synergistic in action in all the spoilage yeasts (FICI ≤ 0.5). It has been reported that vanillin could interact synergistically with membrane targeting antifungals, leading to inhibition of fungal drug efflux pumps [35]. In another study, vanillin led to the disruption of fungal cell surface integrity, dysfunctional mitochondria, and DNA damage when used as a single agent [36]. Cinnamic acid has been proposed as a possible fungal growth inhibitor by interacting with benzoate 4-hydroxylase, crucial for aromatic detoxification [37]. To our knowledge, this is the first study to investigate the antimicrobial effects of vanillin–cinnamic acid combination against food spoilage yeasts.

For the phenolic–synthetic combinations, the prevalence of more synergistic interactions in vanillin–synthetic combinations than in the cinnamic acid–synthetic combinations is attributable to a host of molecular factors. For example, the formation of stable complexes such as dimers and adducts with higher antimicrobial activity than that of the parent compounds, targeting of multiple pathways, modulation of antimicrobial transport and permeation, and inhibition of disease resistance mechanisms [10,32]. A synergic interaction phenomenon has also been ascribed to the recovery of the stronger antioxidant by the weaker antioxidant, as well as the dual targeting of translation fidelity increasing translation error rate thus inhibiting growth [10,34]. It is important to note that synergism effect was not linearly dependent on the concentration of the strongest antimicrobial compound, and in many instances a combination of two high concentrations of the parent compounds did not always have a synergistic effect. In planktonic growth inhibition tests, certain combinations showed different interactions depending on the yeast. For instance, the cinnamic acid and sodium diacetate in combination was antagonistic in action for *D. hansenii* and *W. anomalus* but synergistic in effect in the case of *S. pombe* and additive in action for *S. cerevisiae* (Table 3). These strain-dependent antimicrobial effects of the combinations can also be attributed to the physiological heterogeneity among the different fungi, which also explains the apparent susceptibility of *S. pombe* and the relative resilience of *D. hansenii* against the activity of the combinations. The halotolerant nature of *D. hansenii* has already been reported and characterized [38]. This attribute is attested by the high MIC of the three synthetic preservatives against *D. hansenii* (50 mg/mL) compared to the other tested spoilage yeasts whose MIC of the synthetic preservatives was lower. This peculiarity has been attributed to the adaptability of the plasma membrane components to external salinity, mimicking the halophilic/halotolerant black yeasts [39], and its ability to decrease oxidative stress under hypersaline conditions [40].

The antimicrobial activity of vanillin–synthetic preservatives against *S. cerevisiae* planktonic growth was high (FICI 0.34 and 0.66) (*p* < 0.05). This contrasts with the activity of the cinnamic acid–synthetic preservatives against the same strain, whose FIC indices were 0.9 and 2.82 (*p* < 0.05). Vanillin has been mentioned as a potent inhibitor of the planktonic growth of *S. cerevisiae*, through the disassembly of polysomes and the consequent formation of processing bodies and stress granules, suggesting it acts as a translational repressor in *S. cerevisiae* [41]. Natural phenolics are also known to interfere with the cell membrane integrity, leading to the leakage of cellular organelles and ions, reduction in ergosterol biosynthesis, interference with the cell cycle leading to apoptosis, and inhibition of efflux transporters leading to the accumulation of the antifungal compounds. However, the mechanisms of the interaction of phenolics and other antifungals are less studied [42]. Experiments are mainly focused on interactions with the antifungal drugs generally used against clinical isolates of yeasts [42,43].

Yeast biofilms are resilient structures that require diverse antifungal strategies to obliterate due to the multifactorial nature of antifungal resistance [44]. In fact, fungal cells in biofilms can be up to a thousand-fold more resistant to conventional antimicrobial agents than in planktonic forms [45]. This trait is also manifested in our study where most of the phenolic–synthetic combinations were indifferent against yeast biofilms. However, the vanillin–cinnamic acid combination seems to be an effective strategy. The vanillin–cinnamic acid combination was also effective against yeast adherence on the abiotic surface and might influence the initial stage of biofilm formation.

Vanillin is an aromatic aldehyde with an odor threshold of 0.008 and 100 µg/L in air and water, respectively [46], and is lauded for its appetite-enhancing effects when present in food as an additive [47]. According to the United States Food and Drug Administration (FDA), the maximum permitted level of vanillin is 70 mg/kg of body weight [48]. Cinnamic acid is known for its rapid absorption and elimination from the body and has no safety concerns when used as a flavoring agent [49]. According to the joint FAO/WHO expert committee on food and additives (JECFA), the acceptable daily intake (ADI) levels of sodium diacetate, potassium sorbate, and sodium benzoate is 0–15, 0–25, and 0–5 mg/kg, per body weight, respectively [50,51]. Based on our study, all the combinations with synergistic interactions were within the permitted concentrations of food additives (see Table 1, Table 2, Table 3, Table 4, Table 5, Table 6 and Table 7). All the compounds used in the study are generally recognized as safe (GRAS), therefore, quite appealing for use in food realms.

## 5. Conclusions

The present investigation aimed to elucidate the effect of vanillin–cinnamic acid and vanillin/cinnamic acid–synthetic preservative, i.e., sodium benzoate, potassium sorbate, and sodium diacetate combinations against planktonic and biofilm growth and adhesion of *D. hansenii*, *W. anomalus*, *S. pombe*, and *S. cerevisiae* spoilage yeasts. The outcome of the investigation showed that the vanillin–cinnamic acid combinations were generally more potent antimicrobials than the phenolic–synthetic compound combinations. The mechanism of the dual action of vanillin and cinnamic acid remains to be elucidated; however, chemogenomic profiling and “omics”-based technologies such as transcriptomics and proteomics, as well as systems biology and in silico approaches, may provide insight into the background of the antimicrobial activity achieved in the combined application. In several combinations, the initial MIC of the synthetic preservative was reduced in the presence of a phenolic compound, which may allow the use of a reduced amount of synthetic preservative for food preservation. A phenolic compound-synthetic preservative synergy effect was achieved in anti-planktonic growth and anti-adhesion tests, in which vanillin was the more potent phenolic compound. Among the yeasts studied, planktonic growth of *D. hansenii* was tolerant against the synthetic preservatives both in single and combined application, while the *S. pombe* was generally the most sensitive towards the combinations in anti-biofilm and anti-adhesion tests. The application of phenolics and synthetic compounds in combination could provide a valuable approach for better food preservation without significantly affecting their native organoleptic attributes. Phenolic–phenolic and phenolic–synthetic combinations will also provide new formulations that could attenuate antimicrobial resistance in food spoilage yeasts without the discovery of new anti-yeast molecules. The fact that cinnamic acid is rapidly eliminated from the body is crucial because it could prevent any unfavorable effects due to its accumulation. The finding that all the synergistic combinations were within the permitted levels for food additives allows them to be adopted in food preservation. Though all the compounds used in the study are GRAS, it is a general requirement that compounds permitted for direct addition to food should be used in the minimum quantities required to produce the intended effect. The combinations at which the synergism effect occurred should be tested for the ability to resist the development of antifungal resistance. In addition, the impact of the effective combinations on the organoleptic properties of different foods as well as their chemical compatibility should also be investigated for consumer safety.

## Figures and Tables

**Table 1:** Effect of combination of vanillin and cinnamic acid on planktonic growth of food spoilage yeasts.

Yeasts	Phenolic Compounds	MIC (mg/mL)		MIC Reduction (Fold)	FIC Index	Outcome
		Single	Combined			
*D. hansenii*	Cinnamic acid + Vanillin	0.5 1	≤0.125 ≤0.0625	≥4 ≥16	≤0.31	Synergy
*W. anomalus*	Cinnamic acid + Vanillin	0.5 1	≤0.125 ≤0.0625	≥4 ≥16	≤0.31	Synergy
*S. pombe*	Cinnamic acid + Vanillin	0.5 2	≤0.125 ≤0.0625	≥4 ≥32	≤0.28	Synergy
*S. cerevisiae*	Cinnamic acid + Vanillin	0.5 2	≤0.125 ≤0.0625	≥4 ≥32	≤0.28	Synergy

**Table 2:** Effect of combination of vanillin and synthetic preservatives on planktonic growth of food spoilage yeasts.

Yeasts	Phenolic Agent + Synthetic Preservative	MIC (mg/mL)		MIC Reduction (Fold)	FIC Index	Outcome
		Single	Combined			
*D. hansenii*	Vanillin + Sodium benzoate	1 50	2 1	0.5 50	2.02	Indifferent
	Vanillin + Potassium sorbate	1 50	2 32	0.5 1.56	2.64	Indifferent
	Vanillin + Sodium diacetate	1 50	2 16	0.5 3.13	2.32	Indifferent
*W. anomalus*	Vanillin + Sodium benzoate	1 12.5	0.25 1	4 12.5	0.33	Synergy
	Vanillin + Potassium sorbate	1 3	0.25 1	4 3	0.58	Additive
	Vanillin + Sodium diacetate	1 6	4 4	0.25 1.5	4.67	Antagonism
*S. pombe*	Vanillin + Sodium benzoate	2 6.25	0.5 1	4 6.25	0.41	Synergy
	Vanillin + Potassium sorbate	2 3	0.25 1	8 3	0.46	Synergy
	Vanillin + Sodium diacetate	2 12.5	0.5 2	4 6.25	0.41	Synergy
*S. cerevisiae*	Vanillin + Sodium benzoate	2 25	0.5 2.34	4 10.68	0.34	Synergy
	Vanillin + Potassium sorbate	2 25	0.5 2.34	4 10.68	0.34	Synergy
	Vanillin + Sodium diacetate	2 50	1 8	2 6.25	0.66	Additive

**Table 3:** Effect of combination of cinnamic acid and synthetic preservatives on planktonic growth of food spoilage yeasts.

Yeasts	Phenolic Agent + Synthetic Preservative	MIC (mg/mL)		MIC Reduction (Fold)	FIC Index	Outcome
		Single	Combined			
*D. hansenii*	Cinnamic acid + Sodium benzoate	0.5 50	>4 >64	<0.13 <0.78	>9.28	Antagonism
	Cinnamic acid + Potassium sorbate	0.5 50	0.25 64	2 0.78	1.78	Indifferent
	Cinnamic acid + Sodium diacetate	0.5 50	2 64	0.25 0.78	5.28	Antagonism
*W. anomalus*	Cinnamic acid + Sodium benzoate	0.5 12.5	0.25 1	2 12.5	0.58	Additive
	Cinnamic acid + Potassium sorbate	0.5 3.13	0.13 1	3.85 3.13	0.58	Additive
	Cinnamic acid + Sodium diacetate	0.5 6.25	2 1	0.25 6.25	4.16	Antagonism
*S. pombe*	Cinnamic acid + Sodium benzoate	0.5 6	0.25 1	2 6	0.67	Additive
	Cinnamic acid + Potassium sorbate	0.5 3	0.25 1	2 3	0.83	Additive
	Cinnamic acid + Sodium diacetate	0.5 12.5	≤0.13 ≤1	≥3.85 ≥12.5	≤0.34	Synergy
*S. cerevisiae*	Cinnamic acid + Sodium benzoate	0.5 25	0.13 64	3.85 0.39	2.82	Indifferent
	Cinnamic acid + Potassium sorbate	0.5 25	0.13 64	3.85 0.39	2.82	Indifferent
	Cinnamic acid + Sodium diacetate	0.5 50	0.13 32	3.85 1.56	0.9	Additive

**Table 4:** Effect of combination of phenolic compounds and synthetic preservatives on biofilm formation of food spoilage yeasts.

Yeasts	Phenolic Agent + Synthetic Preservative	MIC (mg/mL)		MIC Reduction (Fold)	FIC Index	Outcome
		Single	Combined			
*W. anomalus*	Vanillin + Sodium benzoate	8 32	16 2	0.5 16	2.06	Indifferent
	Vanillin + Potassium sorbate	8 16	16 1	0.5 16	2.06	Indifferent
	Cinnamic acid + Sodium benzoate	8 32	2 32	4 1	1.25	Indifferent
	Cinnamic acid + Potassium sorbate	8 16	4 32	2 0.5	2.5	Indifferent
*S. pombe*	Vanillin + Potassium sorbate	8 16	16 1	0.5 16	2.06	Indifferent
	Vanillin + Sodium benzoate	8 64	8 2	1 32	1.03	Indifferent
	Vanillin + Sodium diacetate	8 32	4 8	2 4	0.75	Additive
	Cinnamic acid + Sodium diacetate	8 32	4 64	2 0.5	2.5	Indifferent
	Cinnamic acid + Sodium benzoate	8 64	8 1	1 64	1.02	Indifferent
	Cinnamic acid + Potassium sorbate	8 16	8 1	1 16	1.06	Indifferent
*S. cerevisiae*	Vanillin + Potassium sorbate	8 32	16 1	0.5 32	2.03	Indifferent
	Vanillin + Sodium benzoate	8 32	16 1	0.5 32	2.03	Indifferent
	Vanillin + Sodium diacetate	8 64	4 32	2 2	1.00	Indifferent
	Cinnamic acid + Sodium diacetate	8 64	2 64	4 1	1.25	Indifferent

**Table 5:** Effect of combination of vanillin and cinnamic acid on biofilm formation of food spoilage yeasts.

Yeasts	Phenolic Compounds	MIC (mg/mL)		MIC Reduction (Fold)	FIC Index	Outcome
		Single	Combined			
*D. hansenii*	Cinnamic acid + Vanillin	8 8	2 2	4 4	0.5	Synergy
*W. anomalus*	Cinnamic acid + Vanillin	8 8	≤1 ≤0.5	≥8 ≥16	≤0.19	Synergy
*S. pombe*	Cinnamic acid + Vanillin	8 8	1 1	8 8	0.25	Synergy
*S. cerevisiae*	Cinnamic acid + Vanillin	8 8	2 0.5	4 16	0.31	Synergy

**Table 6:** Effect of combination of phenolic compounds and synthetic preservatives on polystyrene surface adhesion of food spoilage yeasts.

Yeasts	Phenolic Agent + Synthetic Preservative	MIC (mg/mL)		MIC Reduction (Fold)	FIC Index	Outcome
		Single	Combined			
*W. anomalus*	Vanillin + Sodium benzoate	8 32	16 1	0.5 32	2.03	Indifferent
	Vanillin + Potassium sorbate	8 16	1 2	8 8	0.25	Synergy
	Cinnamic acid + Sodium benzoate	8 32	4 32	2 1	1.5	Indifferent
	Cinnamic acid + Potassium sorbate	8 16	8 8	1 2	1.5	Indifferent
*S. pombe*	Vanillin + Potassium sorbate	8 8	2 1	4 8	0.38	Synergy
	Vanillin + Sodium benzoate	8 16	4 2	2 8	0.63	Additive
	Vanillin + Sodium diacetate	8 32	4 1	2 32	0.53	Additive
	Cinnamic acid + Sodium diacetate	8 32	4 8	2 4	0.75	Additive
	Cinnamic acid + Sodium benzoate	8 16	4 1	2 16	0.56	Additive
	Cinnamic acid + Potassium sorbate	8 8	4 1	2 8	0.63	Additive
*S. cerevisiae*	Vanillin + Potassium sorbate	8 16	16 1	0.5 16	2.06	Indifferent
	Vanillin + Sodium benzoate	8 16	16 8	0.5 2	2.5	Indifferent
	Vanillin + Sodium diacetate	8 32	8 16	1 2	1.5	Indifferent
	Cinnamic acid + Sodium diacetate	8 32	16 16	0.5 2	2.5	Indifferent

**Table 7:** Effect of combination of vanillin and cinnamic acid on adhesion on polystyrene surface of food spoilage yeasts.

Yeasts	Phenolic Compounds	MIC (mg/mL)		MIC Reduction (Fold)	FIC Index	Outcome
		Single	Combined			
*D. hansenii*	Cinnamic acid + Vanillin	8 8	4 1	2 8	0.63	Additive
*W. anomalus*	Cinnamic acid + Vanillin	8 8	2 0.5	4 16	0.31	Synergy
*S. pombe*	Cinnamic acid + Vanillin	8 8	1 2	8 4	0.38	Synergy
*S. cerevisiae*	Cinnamic acid + Vanillin	8 8	2 2	4 4	0.5	Synergy

## Data Availability

Data are contained within the article and Supplementary Figures S1–S4.

## Supplementary Materials

The following supporting information can be downloaded at: https://www.mdpi.com/article/10.3390/foods12061338/s1, Figure S1: Checkerboard layout of vanillin–cinnamic acid combinations used against the planktonic growth of *D. hansenii* (A), *S. cerevisiae* (B), *S. pombe* (C), and *W. anomalus* (D) spoilage yeasts; Figure S2: Checkerboard layout of phenolic compound–synthetic preservative combinations used against the planktonic growth of *S. pombe* (A and C), *W. anomalus* (B), and *S. cerevisiae* (D); Figure S3: Checkerboard layout of phenolic compound–synthetic preservative combinations used against the biofilm growth of *W. anomalus* (A), *S. pombe* (B and D), and *S. cerevisiae* (C); Figure S4: Checkerboard layout of vanillin–cinnamic acid combinations used against the biofilm growth of *D. hansenii* (A), *W. anomalus* (B), *S. cerevisiae* (C), and *S. pombe* (D).
