# Supplementary material for: Activity of Binary Combinations of Natural Phenolics and Synthetic Food Preservatives against Food Spoilage Yeasts
Source: Foods. 2023 Mar 22;12(6):1338. doi: 10.3390/foods12061338 (PMC10048113; doi:10.3390/foods12061338)
Supplement: Supplementary file 1 [file foods-12-01338-s001.zip › foods-2248735-supplementary.pdf]

Supplementary material

Activity of binary combinations of natural phenolics and synthetic food preservatives against food spoilage yeasts

Bernard Gitura Kimani [1], Miklós Takó [1,*], Csilla Veres [1], Judit Krisch [2], Tamás Papp [1,3], Erika Beáta Kerekes [1,†] and Csaba Vágvölgyi [1,†]

[1] Department of Microbiology, Faculty of Science and Informatics, University of Szeged, Közép fasor 52, H-6726 Szeged, Hungary
[2] Department of Food Engineering, Faculty of Engineering, University of Szeged, Mars tér 7, H-6724 Szeged, Hungary
[3] ELKH-SZTE Fungal Pathogenicity Mechanisms Research Group, University of Szeged, H-6726 Szeged, Közép fasor 52, Hungary

[*]Correspondence: tako78@bio.u-szeged.hu; Tel.: +36-62-544-516
[†]These authors contributed equally to this work.

List of figures

Figure S1. Checkerboard layout of vanillin-cinnamic acid combinations used against the planktonic growth of *D. hansenii* (**A**), *S. cerevisiae* (**B**), *S. pombe* (**C**) and *W. anomalus* (**D**) spoilage yeasts.

Figure S2. Checkerboard layout of phenolic compound-synthetic preservative combinations used against the planktonic growth of *S. pombe* (**A** and **C**), *W. anomalus* (**B**) and *S. cere*visiae (**D**).

Figure S3. Checkerboard layout of phenolic compound-synthetic preservative combinations used against the biofilm growth of *W. anomalus* (**A**), *S. pombe* (**B** and **D**) and *S. cerevisiae* (**C**).

Figure S4. Checkerboard layout of vanillin-cinnamic acid combinations used against the biofilm growth of *D. hansenii* (**A**), *W. anomalus* (**B**), *S. cerevisiae* (**C**) and *S. pombe* (**D**).

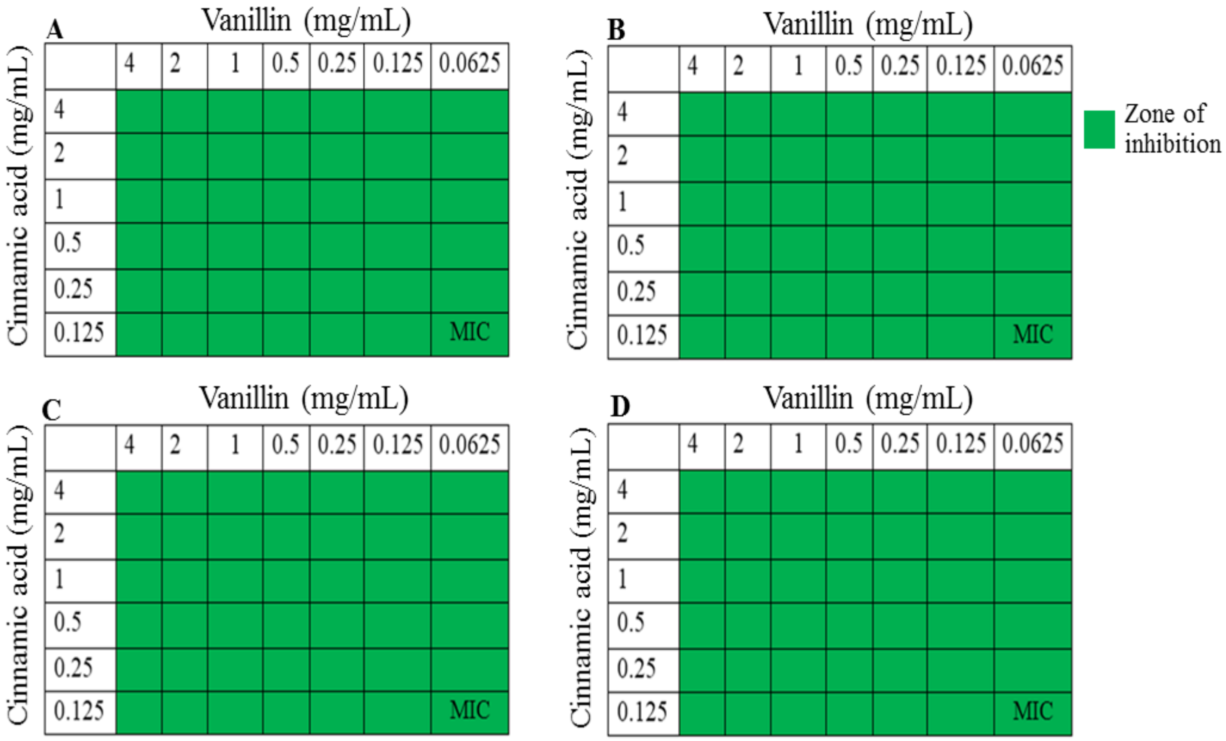

Figure S1. Checkerboard layout of vanillin-cinnamic acid combinations used against the planktonic growth of *D. hansenii* (**A**), *S. cerevisiae* (**B**), *S. pombe* (**C**) and *W. anomalus* (**D**) spoilage yeasts. The MIC is the minimum inhibitory concentration considered in determining the fractional inhibitory concentration index. Lower combinations below the MIC did not inhibit growth.

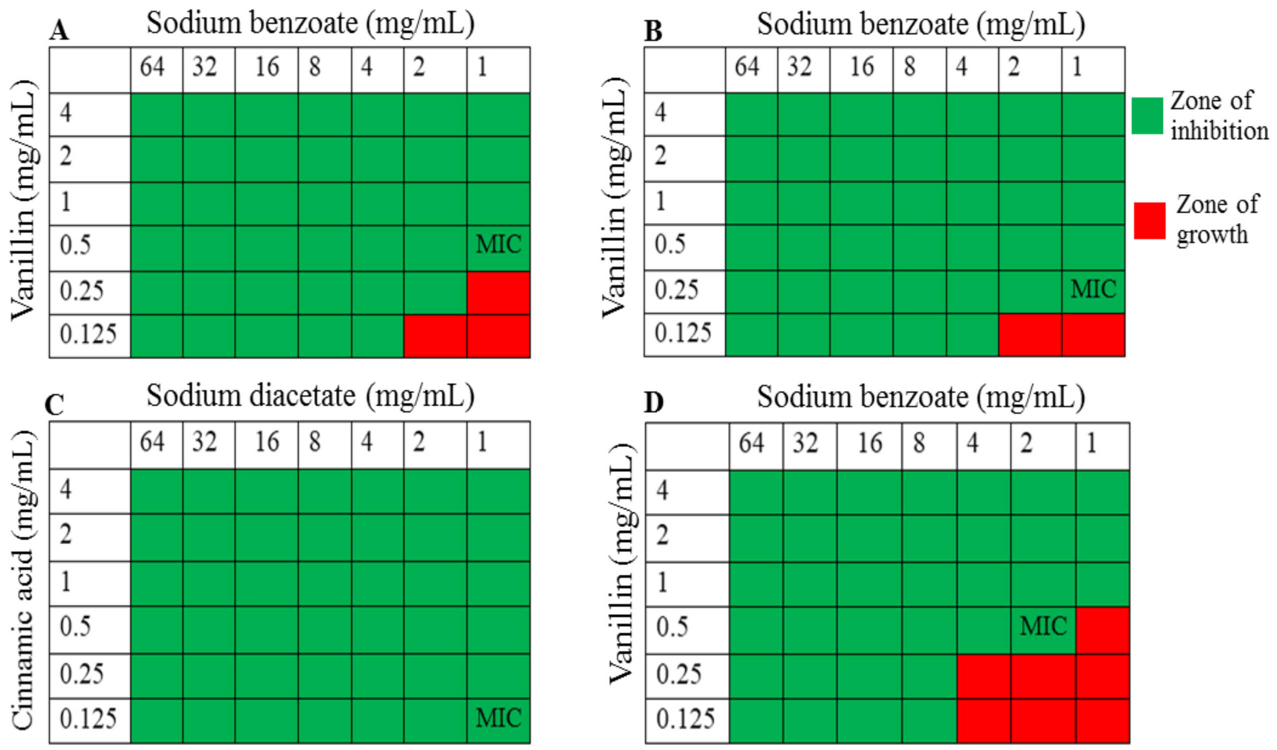

Figure S2. Checkerboard layout of phenolic compound-synthetic preservative combinations used against the planktonic growth of *S. pombe* (**A** and **C**), *W. anomalus* (**B**) and *S. cere*visiae (**D**). The MIC is the minimum inhibitory concentration considered in determining the fractional inhibitory concentration index.

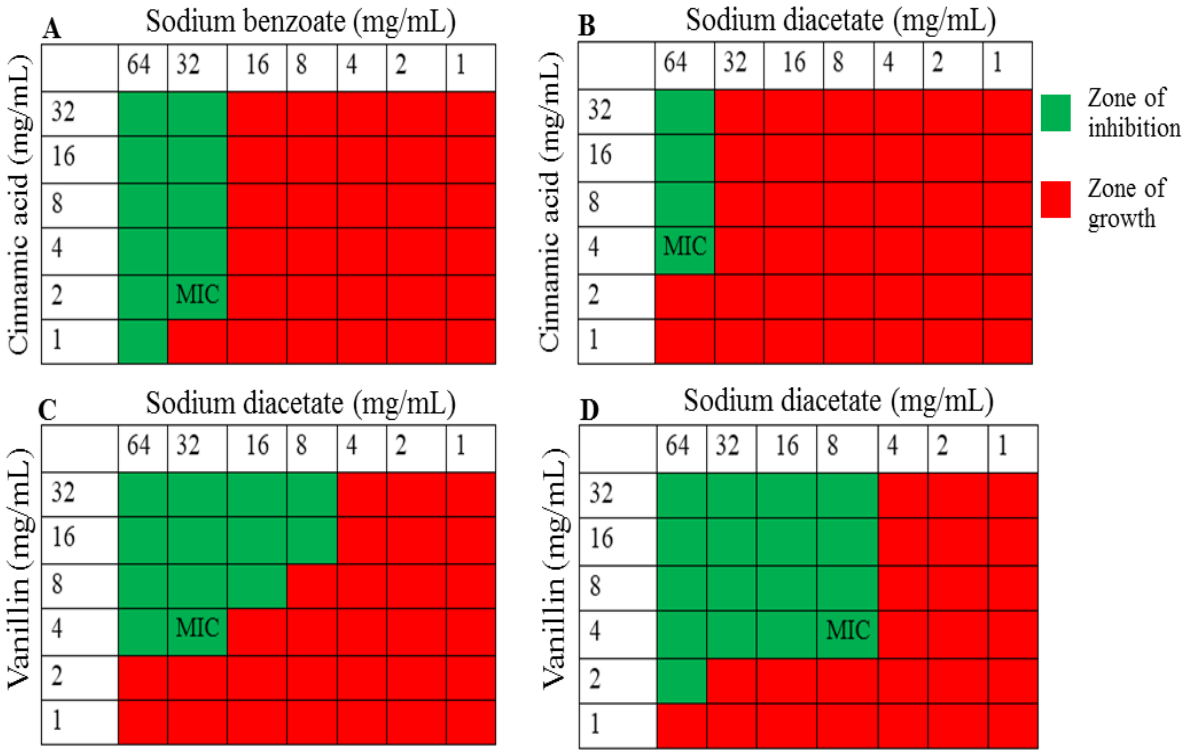

Figure S3. Checkerboard layout of phenolic compound-synthetic preservative combinations used against the biofilm growth of *W. anomalus* (**A**), *S. pombe* (**B** and **D**) and *S. cerevisiae* (**C**). The MIC is the minimum inhibitory concentration considered in determining the fractional inhibitory concentration index.

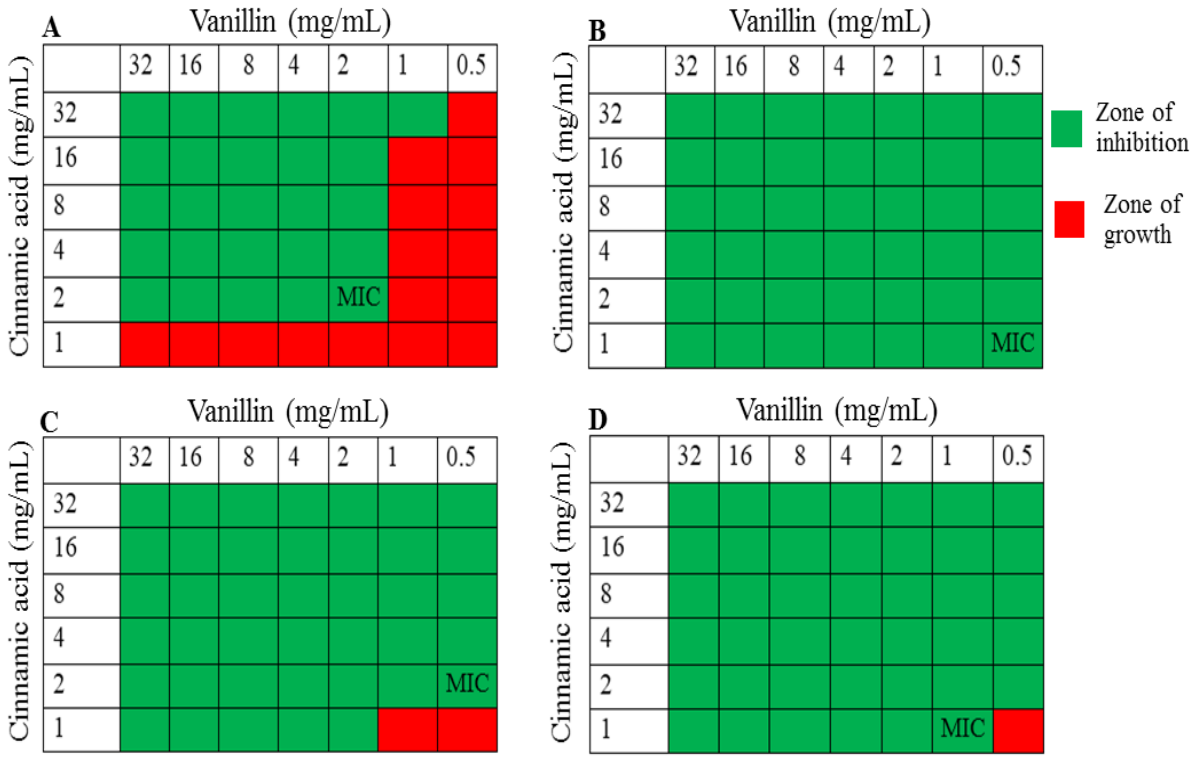

Figure S4. Checkerboard layout of vanillin-cinnamic acid combinations used against the biofilm growth of *D. hansenii* (**A**), *W. anomalus* (**B**), *S. cerevisiae* (**C**) and *S. pombe* (**D**). The MIC is the minimum inhibitory concentration considered in determining the fractional inhibitory concentration index.
